# Nicotinamide mononucleotide promotes osteogenesis and reduces adipogenesis by regulating mesenchymal stromal cells via the SIRT1 pathway in aged bone marrow

**DOI:** 10.1038/s41419-019-1569-2

**Published:** 2019-04-18

**Authors:** Jie Song, Jing Li, Fangji Yang, Gang Ning, Limin Zhen, Lina Wu, Yongyuan Zheng, Qi Zhang, Dongjun Lin, Chan Xie, Liang Peng

**Affiliations:** 1 Department of Infectious Diseases, The Third Affiliated Hospital of Sun Yat-Sen University, Guangzhou, China; 2 Guangdong-Hongkong-Macau Institute of CNS Regeneration, Ministry of Education CNS Regeneration Collaborative Joint Laboratory, Jinan University, Guangzhou, China; 3 Cell-Gene Therapy Translational Medicine Research Center, The Third Affiliated Hospital of Sun Yat-Sen University, Guangzhou, China; 4 Department of Haematology, The Seventh Affiliated Hospital of Sun Yat-Sen University, Shenzhen, China

**Keywords:** Ageing, Stem-cell research

## Abstract

Mesenchymal stromal cells (MSCs) can differentiate to various cell types including osteoblasts, chondrocytes, and adipocytes. This cellular flexibility contributes to widespread clinical use of MSCs in tissue repair. However, challenges remain in efficient cellular expansion of MSCs for stem cell therapy. Current MSC culture methods have resulted in reduced self-renewal of MSCs and compromised therapeutic outcomes. This study identifies that nicotinamide mononucleotide (NMN), a key natural NAD^+^ intermediate, effectively encourages MSC expansion in vitro and in vivo. The in vitro expanded MSCs had heightened osteogenesis, but reduced adipogenesis. Furthermore, NMN supplementation stimulated osteogenesis of endogenous MSCs, and protected bone from aging and irradiation induced damage in mice. Mechanistically, we found that NMN treatment upregulated SIRT1. Genetically overexpressing SIRT1 in MSCs by using *Prx1 cre; ColA1*^*flox-stop-flox-SIRT1*^ mice promoted osteogenesis and reduced adipogenesis in aged mice. Overall, our data demonstrate that NMN promoted MSC self-renewal with strengthened osteogenesis and reduced adipogenesis via upregulating SIRT1 in aged mice.

## Introduction

Aging is predicted to be an increasingly serious health and financial problem worldwide^1^. Age-related disorders, such as tumor^2,3^, metabolic disease^4^, memory deterioration^5^, and immunologic degeneration^6^, are associated with declined regenerative capacity in rapidly dividing stem cells^7^. Nicotinamide mononucleotide (NMN), a key NAD^+^ intermediate which decreases with age in mammals^8^, is an efficient therapy against age-associated diseases^9,10^. NMN administration alleviates age-related type 2 diabetes, ischemia-reperfusion injury, and Alzheimer’s disease^11,12^. However, the underlying mechanism of NMN’s protective effect is still unknown. In this study, we explored NMN’s role in combating age-related disorders via regulating mesenchymal stromal cells (MSCs). MSCs are nonhematopoietic multipotent stem cells with regeneration capacity^13^. Loss in number or functionality of MSCs with age profoundly limits tissue regeneration^14^, However, most current MSC culture methods limits self-renewal potential and functionality of MSCs, leading to compromised therapeutic outcomes^10,15^.

Herein, we have investigated the effects and underlying mechanism of NMN on the expansion and differentiation of mouse MSCs in vitro and in vivo. We have found that NMN promotes MSC self-renewal during in vitro culture and in mice. We have further demonstrated that NMN activates Sirtuin1 (SIRT1), which is an NAD^+^-dependent deacetylase. NMN increases osteogenesis and reduces adipogenesis of MSCs via upregulating SIRT1 in aged mice.

## Materials and methods

### Mice and genotyping

The usage of NMN was according to previous studies^16,17^. The mice used in this study included *Prx1-Cre* mice^18^ and *ColA1*^*flox-stop-flox-SIRT1*^ mice^19^. All mice were backcrossed at least six times onto a C57BL/6J background and housed in the Animal Resource Center of Sun Yat-Sen University. All procedures were approved by the Animal Resource Center of Sun Yat-Sen University Institutional Animal Care and Use Committee. For genotyping of *prx1 cre*^*+*^ mice, the following primers were used: 5′-ATGTCCAATTTACTGA CCGTACA-3′ and 5′-CGCATAACCAGTGAAACAGCATT-3′. For genotyping of *ColA1*^*flox-stop-flox-SIRT1*^ mice, the following primers were used: 5′-TGA CCT CCT CAT TGT TAT TGG A-3′ and 5′-GGC GTG GAG GTT TTT CAG T-3′.

### Bone marrow digestion

The two primary methods used to isolate MSCs from bone marrow (BM), enzymatic digestion, and mechanical isolation. Our pre-experiment results showed that the enzymatic digestion method yields fourfold more MSCs than the mechanical isolation method (Fig. S1A–B). Thus, we chose the enzymatic digestion method to acquire MSCs. The enzymatic digestion method was described previously^13,16^. Intact marrow plugs were flushed from the femur and treated with digestion buffer containing 3 mg/ml type I collagenase (Worthington, USA), 4 mg/ml dispase (Roche Diagnostic, USA) in HBSS with Ca^2+^ and Mg^2+^.

### Colony-forming unit-fibroblast (CFU-F) culture and differentiation

CFU-F culture was performed as described previously^20,21^. Briefly, cells acquired by the enzymatic digestion were seeded at a clonal density of 2.5 × 10^5^ cells per well in six-well plates with DMEM (Gibco, USA) plus 10% fetal bovine serum (Gibco, USA), 10 mM ROCK inhibitor (Tocris, UK), and 1% penicillin/streptomycin (Invitrogen, USA).

### Flow cytometry

The antibodies used to analyse MSCs included anti-CD45-APC (Clone 30-F11, 1:100; BioLegend, USA), anti-Ter119-APC (Clone TER-119, 1:100; BioLegend, USA), anti-CD31-PE-cy7 (Clone MEC13.3, 1:100; BioLegend, USA), anti-PDGFRα-biotin (Clone APA5, 1:200; eBioscience, USA), PE anti-mouse CD51 antibody (Clone RMV-7, 1:100; eBioscience, USA), anti-LepR-biotin antibody (BAF497,1:100; R&D Systems, USA), and Anti-Sca1-Percp-cy5.5 (eBioscience, USA).

### MSCs differentiation in culture

MSCs acquired by the enzymatic digestion method were seeded at a clonal density of 2.5 × 10^5^ cells per well in six-well plates to form primary CFU-Fs for 1 week followed by adipogenic (1 week) or osteogenic (2 weeks) differentiation with StemPro Differentiation Kits (Invitrogen, USA).

### MicroCT analysis

The procedure was performed according to previous studies^20,22^.

### Sublethal irradiation

For sublethal irradiation, C57BL/6J mice were X-ray irradiated with an XRAD 320 irradiator (Precision X-Ray, Inc.) at the dosage of 300 rad. Mice were maintained on antibiotic water for 14 days after irradiation.

### Bone sectioning and staining

Dissected bones were fixed in 4% paraformaldehyde overnight, decalcified in 10% EDTA for 48 h and dehydrated in 30% sucrose for 2 days. Bones were sectioned (20 μM thickness) using a slicer (Thermo Scientific, USA). Sections were stained with an Oil Red S or Alizarin Red staining kit.

### qPCR

For quantitative reverse transcription PCR, RNA was extracted, and reverse transcribed into cDNA using SuperScript III (Invitrogen, USA). qPCR was performed using a Roche LightCycler 480. The primers used for qPCR analysis included Runx2 (NM_001146038.2): 5′-TTA CCT ACA CCC CGC CAG TC-3′ and 5′-TGC TGG TCT GGA AGG GTC C-3′; Wnt4: 5′-CCGGGCACTCATGAATCT-3′ and 5′-CACGCCAGCACGTCTTTAC-3′; Adipq: 5′-TGTTCCTCTTAATCCTGCCCA-3′ and 5′- CCAACCTGCACAAGTTCCCTT-3′; and Pparg: 5′-ACCACTCGCATTCCTTTGAC-3′ and 5′-TGGGTCAGCTCTTGTGAATG-3′.

### Immunoblot analysis

The procedure was performed according to previous studies^23^. Proteins were detected using commercially available antisera (α-Tubulin Sigma, USA; SIRT1: Millipore, USA).

### Caspase-3/7 activity measurement

Osteoblasts (CD45^−^Ter119^−^CD31^−^Sca1^−^CD51^+^) were sorted from BM cells and seeded into 48-well plates at a density of 1.5 × 10^4^ per well^24^. Apoptotic cells were detected by the Cell Event Caspase-3/7 Green Detection Reagent (Invitrogen, USA) according to the manufacturer’s instructions. The cells were incubated with detection reagent for 30 min, followed by fixation and quantification.

### EdU incorporation

MSCs or osteoblasts were sorted from BM cells and then seeded into 48-well plates at a density of 1.5 × 10^4^ per well. EdU was added to the MSCs medium at day 0 (10 µM final concentration) and maintained for 48 h^13^. Cell proliferation was detected by the EdU Cell Proliferation Assay Kit (RiboBio, Guangzhou, China) according to the manufacturer’s instructions. Cells were captured using a Leica fluorescent microscope equipped with a camera.

### Statistical analysis

The statistical significance of differences between the two treatments was assessed using two-tailed Student’s *t*-test. The statistical significance of differences among groups was assessed using one-way ANOVA with Tukey’s multiple comparison tests. The statistical significance of differences in long-term competitive reconstitution assays was assessed using two-way ANOVA with Sidak’s multiple comparison tests. All data represent the mean ± SD. *p < 0.05, **p < 0.01, ***p < 0.001.

## Results

### NMN promotes MSC expansion in vitro and in adult mice

**Fig. 1:**
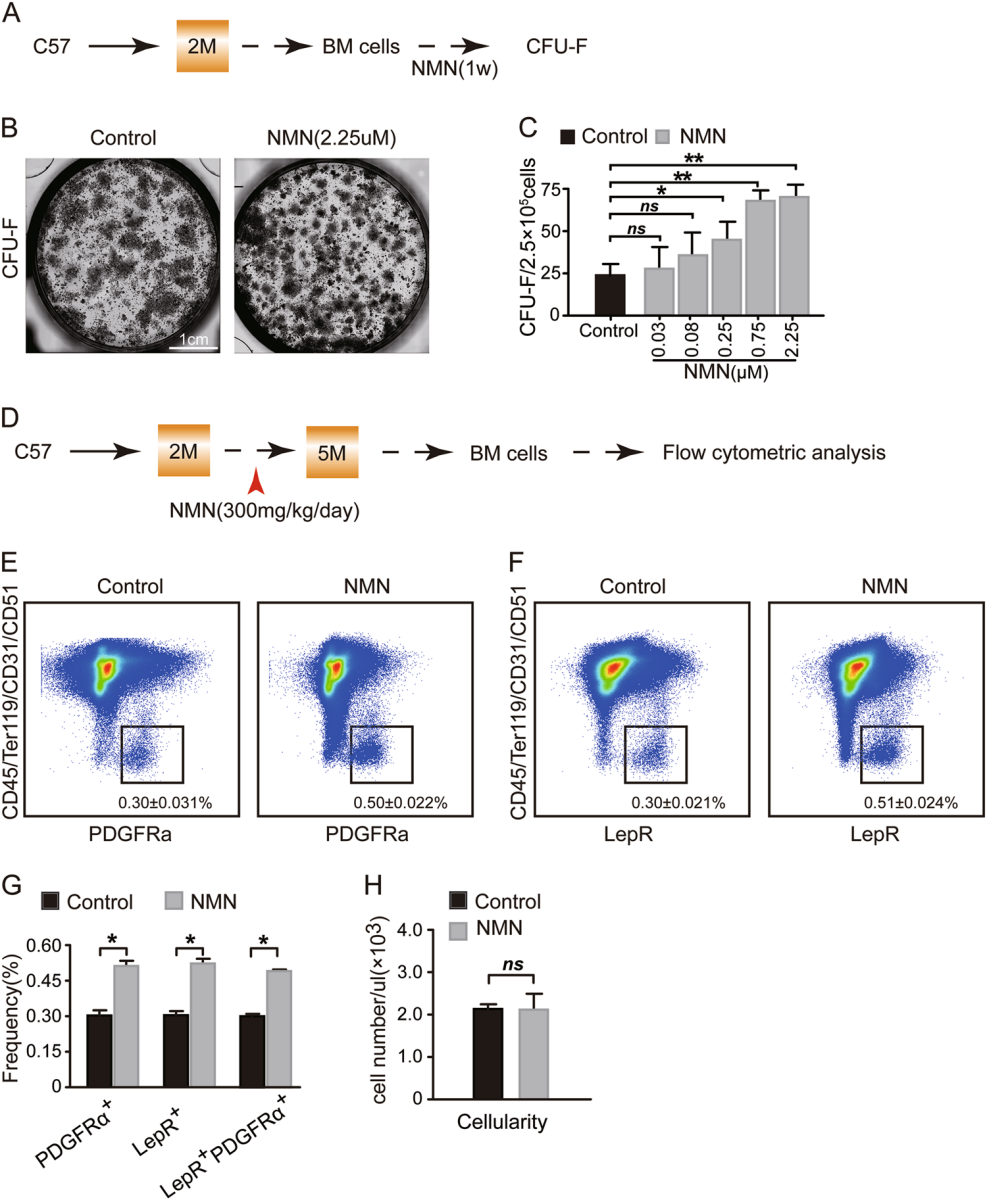
NMN promotes MSC expansion in an in vitro culture system and in adult mice. **a** Scheme of NMN administration and the CFU-F assay. **b** Representative images acquired from live cell imaging system showing CFU-F colonies formed by MSCs. **c** The number of CFU-F contained in MSCs. **d** Scheme of NMN administration and flow cytometric analysis. **e**, **f** The PDGFRα^+^ MSCs (**e**), and LepR^+^ MSCs (**f**) from enzymatically dissociated femur bone marrow cells are shown. **g** The frequencies of LepR^+^ MSCs, PDGFRα^+^ MSCs, and PDGFRα^+^ LepR^+^ MSCs from enzymatically dissociated bone marrow cells. **h** The cellularity of the enzymatically dissociated bone marrow cells (*n* = 6 mice per group from three independent experiments). *p < 0.05, **p < 0.01 error bars, s.d.

To investigate the effects of NMN on the expansion of MSCs in vitro, we performed the CFU-F assay using enzymatically digested mouse BM cells administered with NMN (Fig. 1a). The results showed that the CFU-F frequency increased in response to NMN administration in a dosage dependent manner (0.08–2.25 μM) (Fig. 1b, c) and reached the maximum at the concentrations between 0.75 and 2.25 μM. NMN treatment at 0.03 and 0.08 μM did not show significant change in CFU-F frequency (Fig. 1c). These results indicate that NMN promotes MSC expansion and may be a useful tool to enhance MSC frequency in vivo.

To investigate if NMN stimulates MSCs expansion in vivo, we analysed immunophenotypic MSCs^13,20^ (PDGFRα^+^CD45^−^Ter119^−^CD31^−^CD51^+^ stromal cells or LepR^+^CD45^−^Ter119^−^CD31^−^CD51^+^ stromal cells) from mice with NMN administration (Fig. 1d). We found that the MSCs frequency increased to 0.50 ± 0.022% 3 months after NMN treatment (Fig. 1e–g) compared to the control group (0.30 ± 0.031%). Similarly, the frequency of LepR^+^CD45^−^Ter119^−^CD31^−^CD51^+^ stromal cells increased to 0.51 ± 0.024% (Fig. 1f, g) compared to the control group (0.30 ± 0.021%). Over 95% of LepR^+^CD45^−^Ter119^−^CD31^−^CD51^+^ stromal cells were positive for PDGFRα and vice versa (Figs. S1C and 1g). However, we did not observe cellularity change in response to NMN treatment (Fig. 1h). Altogether, our results suggest that NMN significantly encourages MSC expansion in vitro and in vivo.

### Enhanced osteogenesis, but reduced adipogenesis in NMN-treated MSCs in vitro

**Fig. 2:**
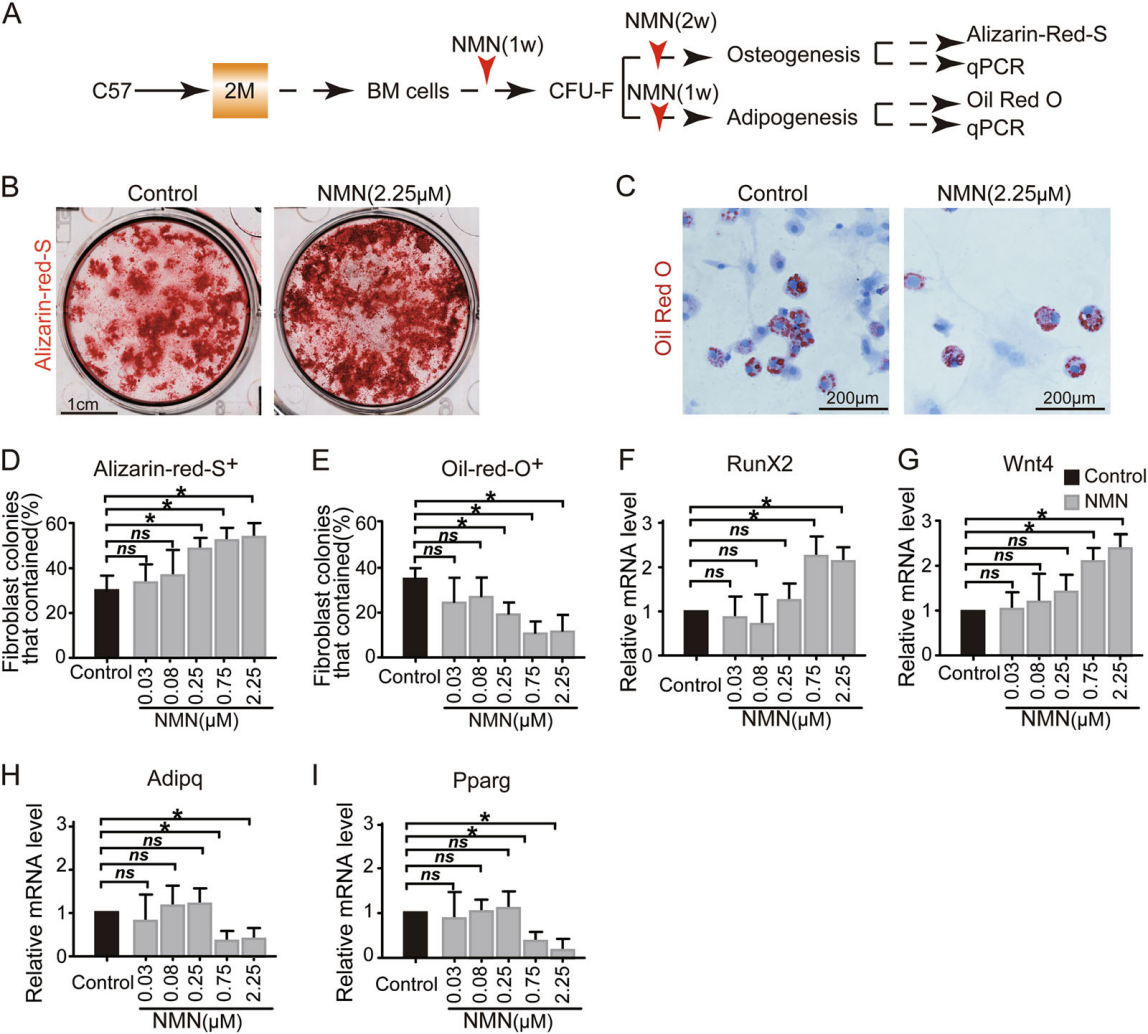
NMN expanded MSCs have enhanced osteogenesis but reduced adipogenesis capacity in an in vitro culture system. **a** Scheme of NMN administration and osteogenesis and adipogenesis. **b**, **c** Representative images showing osteogenesis (**b**) and adipogenesis (**c**). **d**, **e** The percentages of fibroblast colonies that contained osteogenetic (**d**) and adipogenetic (**e**) differentiation of CFU-F. **f**–**i** qPCR analysis of transcript levels for regulators of osteogenic (**f**, **g**) and adipogenic (**h**, **i**) differentiation are shown (*n* = 3 independent experiments). *p < 0.05, **p < 0.01 error bars, s.d.

To investigate the effects of NMN on MSCs differentiation in vitro, we induced CFU-F colonies to osteoblast and adipocyte lineages with NMN administration during differentiation (Fig. 2a). We found significantly more osteoblasts (~50% Alizarin R^+^ colonies) in the culture treated with NMN compared with ~30% in the control group (Fig. 2b, d). Moreover, we found a dosage dependent (0.03–2.25 μM) decrease in Oil Red O^+^ colonies with ~10% in NMN-treated cells and ~37% from control cells (Fig. 2c, e). We observed a substantial decrease of adipocyte differentiation in cells treated with NMN at concentrations between 0.75 (70% of Oil Red O^+^ colonies) and 2.25 µM (68% of Oil Red O^+^ colonies). These results are consistent with previous findings that the anti-aging compound resveratrol stimulated osteogenic differentiation, but inhibited adipogenic differentiation in human embryonic stem cell-derived mesenchymal progenitors^25^.

Our qPCR analysis of NMN-treated cells (0.75 to 2.25 µM) showed significantly increased expression of *RunX2* (Fig. 2f), a critical transcription factor that promotes osteogenesis^26^. Similarly, the expression of *Wnt4*, a noncanonical Wnt ligand that promotes osteogenesis^27^, increased in the NMN-treated group (0.75–2.25 µM) (Fig. 2g). In contrast, the expression level of key adipogenesis transcription factors such as *Pparg* and *Adipq*^28^, declined after NMN treatment (0.75 and 2.25 µM) (Fig. 2h, i).

### NMN does not change bone-fat balance in adult mice

**Fig. 3:**
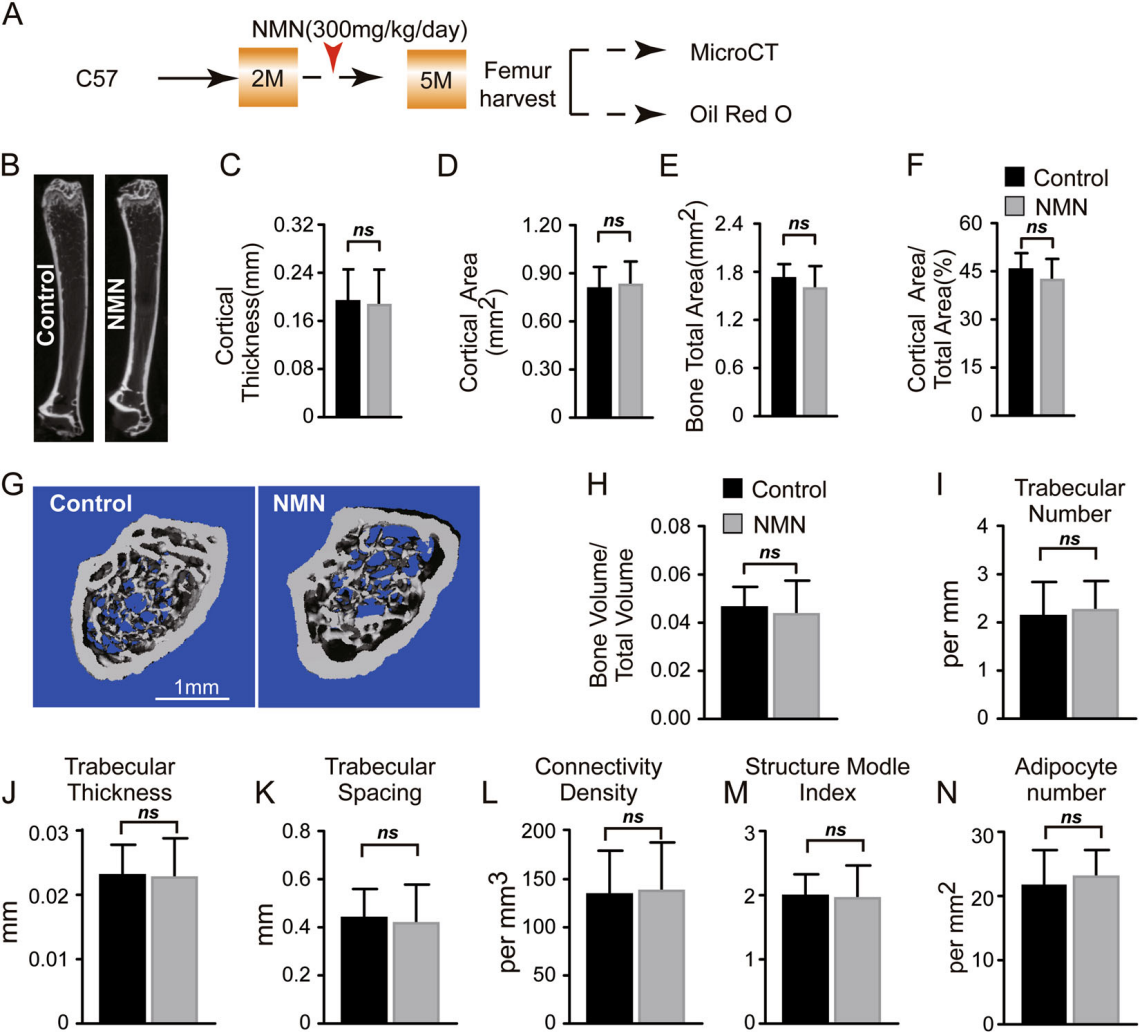
NMN does not affect osteogenesis and adipogenesis in adult mice. **a** Scheme of NMN administration followed by µCT and Oil Red O staining. **b** Representative µCT images showing vertical sections whole femur of 5-month-old NMN-treated or control mice. **c**–**f** µCT analyses of the cortical bone thickness (**c**), cortical bone area (**d**), total area (**e**), and cortical bone area/total area (**f**) (*n* = 6 mice per group, from six independent experiments). **g** Representative µCT images showing the trabecular bone. **h**–**m** µCT analyses of the trabecular bone volume/total volume (**h**), trabecular number (**i**), trabecular thickness (**j**), trabecular spacing (**k**), connectivity density (**l**), and structure model index (**m**) (*n* = 6 mice per group, from six independent experiments). **n** Quantification of the adipocyte number (*n* = 6 mice per group from six independent experiments). *p < 0.05, **p < 0.01 error bars, s.d.

We further explored the effects of NMN on osteogenesis and adipogenesis in vivo by administering NMN to 2-month-old mice for 3 months (Fig. 3a). A micro-computed tomography (µCT) analysis of morphological parameters revealed that thickness, bone volume, and bone area of neither cortical nor trabecular bone changed in the femur of NMN-treated mice compared to controls (Fig. 3b–m). These results indicate that NMN does not affect osteogenesis in adult mice.

To test the effects of NMN on generation of marrow adipose tissue (MAT) in adult mice, we prepared thick femur sections (20 µM) and stained them with Oil Red S. We did not find any significant difference between NMN-treated and control mice (Fig. 3n). These data suggest that NMN does not affect adipogenesis in adult mice.

### NMN potentiates bone accumulation and MAT loss in aged mice

**Fig. 4:**
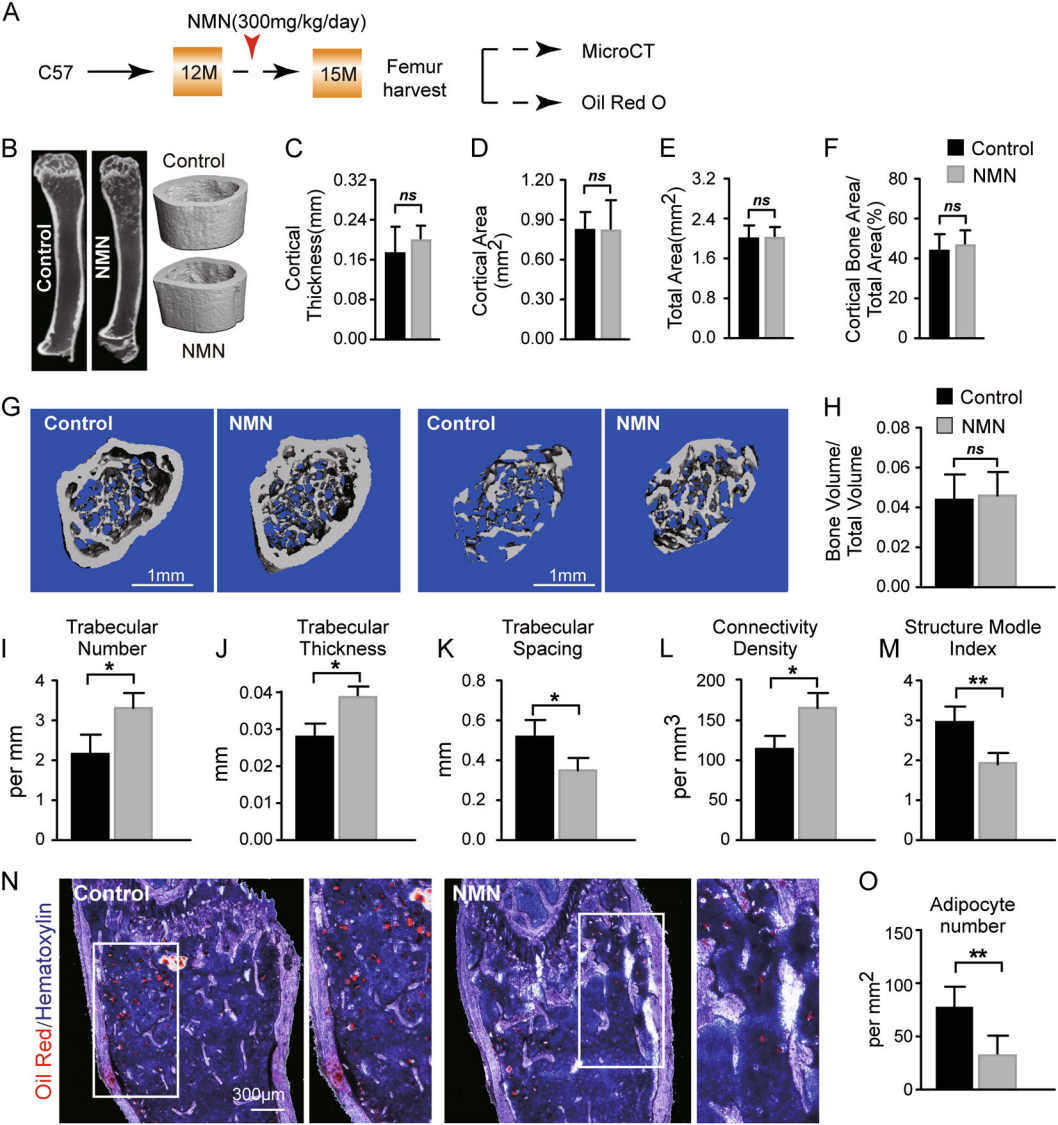
NMN increases osteogenesis but decreases adipogenesis in aged mice. **a** Scheme of NMN administration followed by µCT and Oil Red O staining. **b** Representative µCT images showing vertical sections of whole femur of 15-month-old NMN-treated or control mice. **c**–**f** µCT analyses of the cortical bone thickness (**c**), cortical bone area (**d**), total area (**e**), and cortical bone area/total area (**f**) are shown (*n* = 6 mice per group, total, from six independent experiments). **g** Representative µCT images showing the trabecular bone. **h**–**m** µCT analyses of the trabecular bone volume/total volume (**h**), trabecular number (**i**), trabecular thickness (**j**), trabecular spacing (**k**), connectivity density (**l**), and structure model index (**m**) are (*n* = 6 mice per group from six independent experiments). **n**–**o** Representative Oil Red O and hematoxylin staining in femur sections (**n**) and quantification of the adipocyte number (**o**) (*n* = 6 mice per group from six independent experiments). *p < 0.05, **p < 0.01 error bars, s.d.

Skeletal aging and osteoporosis occur when marrow MSCs aberrantly differentiate towards adipose lineage at the cost of osteogenesis, leading to compromised bone formation and loss of bone mass^29,30^. Hence, we investigated the effects of NMN on lineage specification of marrow MSCs in aged mice by administrating NMN to 12-month-old mice (Fig. 4a). Our µCT analysis revealed that cortical bone parameters in the femur diaphysis of the NMN treatment group were statistically nondistinct to the control group (Fig. 4b–f). In contrast, the trabecular bone gained ~1 per mm in number, ~0.01 mm in thickness, ~48% in trabecular bone connectivity density. Consistently, NMN treatment also decreased trabecular spacing by ~36% and structure model index by ~33% (Fig. 4g–m). Bone volume (BV/TV) did not change in the femur metaphysis of NMN-treated mice compared to control (Fig. 4h). These results indicate that NMN stimulates osteogenesis in aged mice.

To test the implication of NMN on adipogenesis in aged mice, we quantified MAT after NMN treatment. Compared to 75 adipocytes per mm^2^ in the control group, we observed significantly fewer adipocytes (30 adipocytes per mm^2^) in the femur sections from NMN-treated mice than the control (Fig. 4n, o). These data suggest that NMN inhibits adipogenesis in aged mice. Taken together, we demonstrated biased differentiation of MSCs towards bone lineage at the expense of adipocyte lineage after NMN treatment.

### NMN facilitates the expansion of BM MSCs in irradiated adult mice

**Fig. 5:**
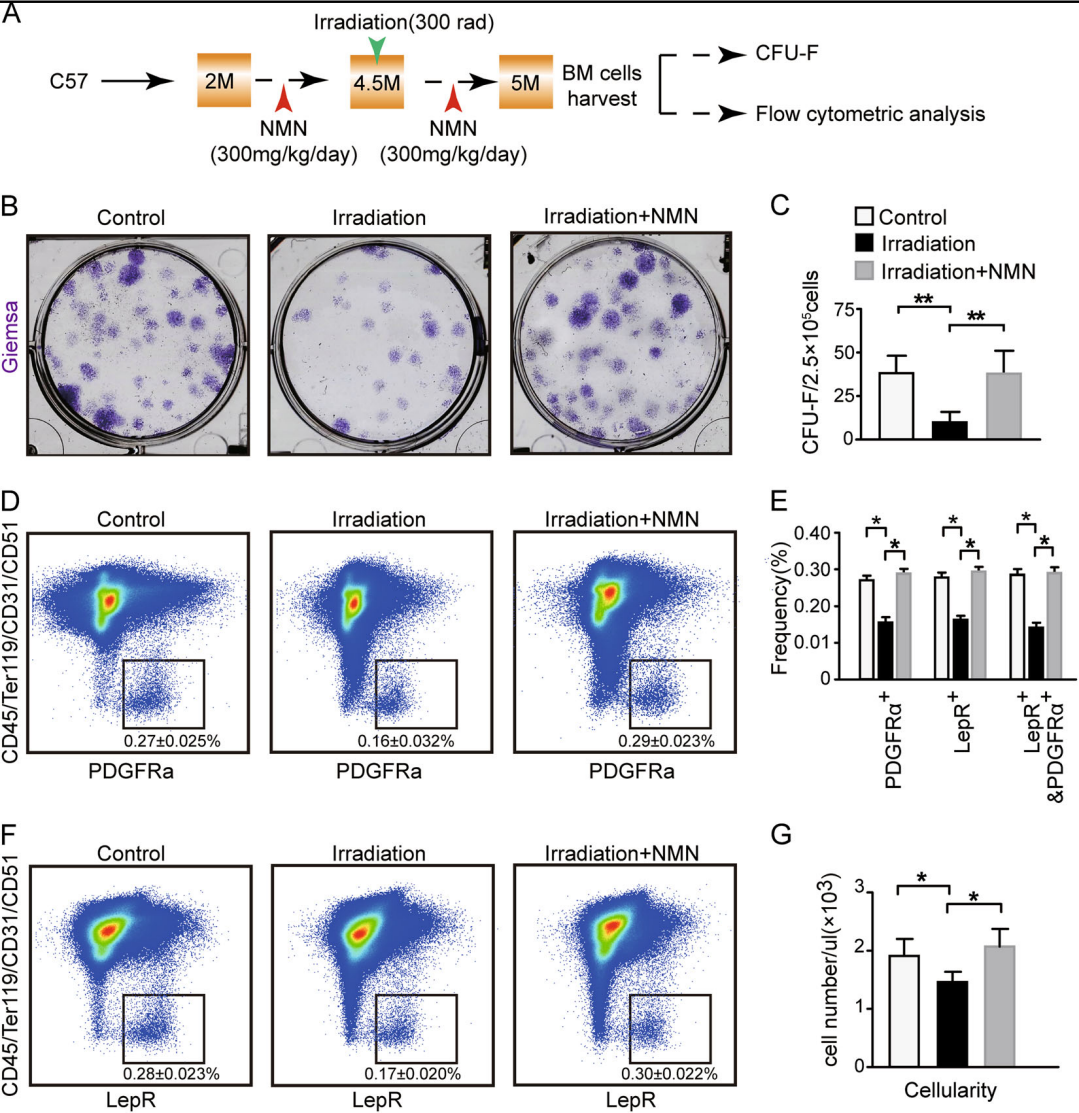
NMN increases the expansion of BM MSCs in irradiated adult mice. **a** Scheme of NMN administration followed by CFU-F and flow cytometry. **b**–**c** Representative images (**b**) and number of CFU-F colonies (**c**) formed by MSCs. **d**–**f** The PDGFRα^+^ MSCs (**d**) and LepR^+^ MSCs (**f**) from enzymatically dissociated femur bone marrow cells. **e** The frequency of LepR^+^ MSCs, PDGFRα^+^ MSCs, and PDGFRα^+^ LepR^+^ MSCs from enzymatically dissociated femur bone marrow cells. **g** The cellularity of the enzymatically dissociated femur bone marrow cells (*n* = 6 mice per group, from three independent experiments). *p < 0.05, **p < 0.01 error bars, s.d.

Since ionizing irradiation often leads to bone loss and damage to MSCs^31,32^, we evaluated the protection of NMN against irradiation of MSCs (Fig. 5a). We found that the CFU-F ability of MSCs from irradiated mice was decreased by ~75% compared to the control group. The NMN treatment restored the CFU-F ability of the irradiated MSCs to the level of the control group (Fig. 5b, c). These results suggest that NMN protects MSCs after irradiation in vitro.

To investigate the protection of NMN on MSCs against irradiation in vivo, we measured the frequency of PDGFRα^+^CD45^−^Ter119^−^CD31^−^CD51^+^ stromal cells (Fig. 5d), LepR^+^CD45^−^Ter119^−^CD31^−^CD51^+^ stromal cells (Fig. 5f), and LepR^+^PDGFRα^+^CD45^−^Ter119^−^CD31^−^CD51^+^ stromal cells (Fig. S2A). We found that the frequency of the above three subsets of MSCs from the irradiation group were decreased by ~41% compared to the control group but were restored after NMN treatment to the level of the control group (Fig. 5d–f). Moreover, the number of total nucleus cells (TNCs) slightly dropped in the irradiation group by ~25% compared to the control group (Fig. 5g) but was again restored to the level of the control group after NMN treatment (Fig. 5g). Furthermore, we found that irradiation inhibited EdU^+^ proliferating MSC by 50%; and the inhibition on MSC proliferation was fully rescued by NMN treatment in irradiated mice (Fig S2B–D). These results suggest that NMN is critical in protecting MSCs from irradiation induced exhaustion by promoting MSCs expansion.

### NMN potentiates bone accumulation and MAT loss in adult mice upon irradiation

**Fig. 6:**
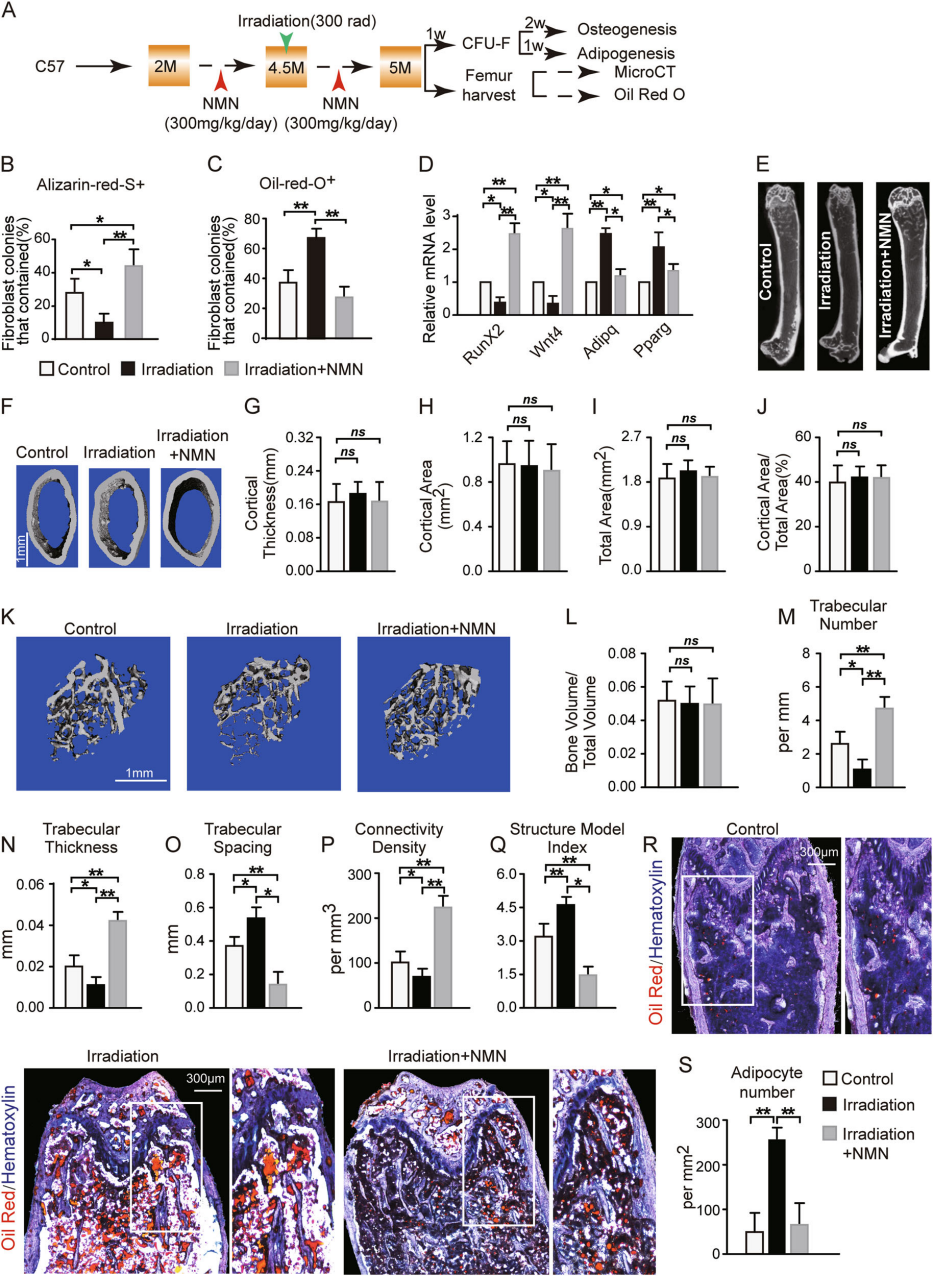
NMN increases osteogenesis but decreases adipogenesis in adult mice under irradiation. **a** Scheme of NMN administration and downstream assays. **b**–**c** The percentage of fibroblast colonies that contained osteogenesis (**b**) and adipogenesis (**c**) differentiation of CFU-Fs are **d** qPCR analysis of expression for regulatory genes of osteogenic and adipogenic differentiation in LepR^+^CD45^−^Ter119^−^CD31^−^ cells from 5-month-old NMN-treated and control mice (*n* = 3 independent experiments). **e**–**f** Representative µCT images showing vertical (**e**) and cross (**f**) sections of whole femur. **g**–**j** µCT analyses of the cortical bone thickness (**g**), cortical bone area (**h**), total area (**i**), and cortical bone area/total area (**j**) in the femur metaphysis (*n* = 6 mice per group from six independent experiments). **k** Representative µCT images showing trabecular bone from the femur metaphysis. **l**–**q** µCT analyses of the percentage of trabecular bone volume out of total volume (**l**), trabecular number (**m**), trabecular thickness (**n**), trabecular spacing (**o**), connectivity density (**p**), and structure model index (**q**) in the femur metaphysis (*n* = 6 mice per group from six independent experiments). **r**–**s** Representative Oil Red S and hematoxylin staining in femur sections (**r**) and quantification of the adipocyte number (**s**) (*n* = 6 mice per group from six independent experiments). *p < 0.05, **p < 0.01 error bars, s.d.

Exposure to sublethal irradiation led to fewer osteoblast but more adipocyte derived from LepR^+^CD45^−^Ter119^−^CD31^−^CD51^+^ stromal cells in bone marrow^13^. Thus, we further assessed the effects of NMN on osteogenesis and adipogenesis of MSCs under irradiation in vitro and in vivo (Fig. 6a). The frequency of Alizarin-Red-S^+^ osteoblastic cells increased by approximately threefold after NMN treatment (Fig. 6b). Meanwhile, Oil-red-O^+^ adipocytes doubled after irradiation, whereas NMN treatment inhibited the growth of MAT (Fig. 6c). Interestingly, mRNA expression of the key osteogenic genes *RunX2* and *Wnt4* decreased by ~50% with irradiation compared to the control, and NMN treatment lead to fivefold enhanced expression of these genes (Fig. 6d). In addition, mRNA expression of adipogenic genes *Adipq* and *Pparg* increased with irradiation by approximately twofold but was inhibited in response to NMN treatment.

Next, we evaluated the effects of NMN on osteogenesis and adipogenesis of MSCs in vivo by µCT and Oil Red S staining in femur sections after irradiation (Fig. 6a). µCT analysis showed that the cortical bone area and thickness did not change after irradiation or NMN treatment (Fig. 6f–j). No percentile change was detected in trabecular bone volume in response to NMN treatment (Fig. 6l). In contrast, we observed decreased trabecular number by ~50%, trabecular thickness by ~30%, and trabecular bone connectivity density by ~20% (Fig. 6m–p), as well as increased trabecular spacing by ~30% and structure model index by ~50% after irradiation (Fig. 6o–q). Importantly, NMN treatment reversed the changes in the above morphological parameters in trabecular bone after irradiation (Fig. 6m–q). These results suggest that NMN promotes post irradiation osteoblast recovery in vivo, particular in the trabecular bone.

Furthermore, NMN treatment effectively inhibited the post irradiation adipogenesis by ~66% (Fig. 6r, s). Altogether, these results confirm our hypothesis that NMN supplementation encourages osteogenesis, but inhibits adipogenesis of MSCs after irradiation. We also noticed that osteoblast cells from irradiated mice have slightly increased (by 8%) proliferation and reduced (by 6%) apoptosis rate after NMN treatment in vitro (Fig. S3). This suggested that NMN might have direct effect on osteoblast cells in irradiated mice.

### NMN controls bone-fat balance of MSCs differentiation through SIRT1

**Fig. 7:**
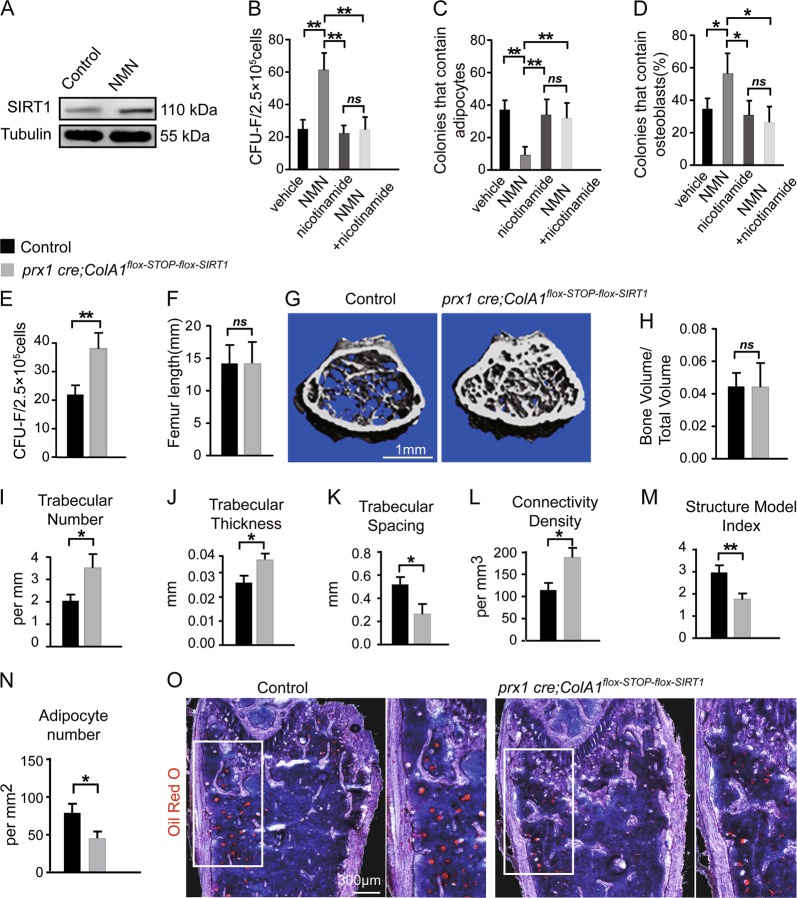
NMN increased osteogenesis but decreased adipogenesis of BM MSCs via a SIRT1-dependent pathway. **a** As shown in Fig. S4A, representative immunoblot showing SIRT1 expression in LepR^+^CD45^−^Ter119^−^CD31^−^CD51^+^ MSCs from the femur of 15-month-old mice supplemented with NMN (300 mg/kg/day, in drinking water) for 3 months compared with the control (*n* = 6 mice per group from three independent experiments). **b**–**d** As shown in Fig. S4B, clonal differentiation of MSCs from 15-month-old mice with or without a SIRT1 inhibitor (nicotinamide, 10 mM) and/or 0.75 μM NMN, for 1 week during CFU-F formation (**b**), another 1 week during adipogenic differentiation (**c**) or another 2 weeks during osteogenic differentiation (**d**) (*n* = 6 mice per group from three independent experiments). **e**–**o** As shown in Fig. S4C, parameters of femurs from 15-month-old *Prx1 cre; ColA1*^*flox-stop-flox-SIRT1*^ and littermate control mice (*n* = 6 mice per group from six independent experiments). **e** CFU-F frequency of MSCs from *Prx1 cre*; ColA1^flox-stop-flox-SIRT1^ and littermate control mice. **f** Femur length of MSCs from *Prx1 cre*; ColA1^flox-stop-flox-SIRT1^ and littermate control mice. **g** Representative µCT images showing trabecular bone in the femur metaphysis of *Prx1 cre; ColA1*^*flox-stop-flox-SIRT1*^ and littermate control mice. **h**–**m** µCT analyses of the percentage of trabecular bone volume out of total volume (**h**), trabecular number (**i**), trabecular thickness (**j**), trabecular spacing (**k**), connectivity density (**l**), and structure model index (**m**) in the femur metaphysis of *Prx1 cre; ColA1*^*flox-stop-flox-SIRT1*^ and littermate control mice. **n**, **o** Quantification of adipocyte number (**n**) and representative Oil Red S and hematoxylin staining in femur sections from *Prx1 cre; ColA1*^*flox-stop-flox-SIRT1*^ and littermate control mice (**o**). *p < 0.05, **p < 0.01 error bars, s.d.

Next, we investigated the mechanism underlying NMN regulation of osteogenesis and adipogenesis in MSCs. SIRT1 promotes osteogenesis and inhibit adipogenesis^33^. We found approximately twofold increase of SIRT1 protein in sorted LepR^+^CD45^−^Ter119^−^CD31^−^CD51^+^ stromal cells from 12-month-old mice treatment with NMN (Fig. 7a and S4A). We further tested whether SIRT1 is required for the effects of NMN on MSCs in 15-month-old mice (Fig. S4B). The CFU-F frequency and osteogenic capacity of MSCs in vitro were supressed, whereas the adipogenic capacity was derepressed by the SIRT1 inhibitor nicotinamide (Fig. 7b–d). These results suggest that SIRT1 is crucial for NMN to promote osteogenesis and inhibit adipogenesis of MSCs.

To confirm our in vitro findings, we investigated osteogenesis and adipogenesis using the *Prx1 cre; ColA1*^*flox-stop-flox-SIRT1*^ mice (Fig. S4C), which overexpresses SIRT1 protein in limb MSCs. We observed that cortical bone parameters in the femur diaphysis of *Prx1 cre; ColA1*^*flox-stop-flox-SIRT1*^ mice were similar to their littermate controls (data not shown). Interestingly, although neither femur length nor the trabecular bone volume changed in *Prx1 cre; ColA1*^*flox-stop-flox-SIRT1*^ mice (Fig. 7f–h), we detected doubled CFU-F frequency in enzymatically isolated BM (Fig. 7e), which was potentially caused by overall strengthened trabecular bone in epiphysis region including substantially denser trabeculae structure, increased trabecular number by ~60%, thickness by ~25%, and trabecular bone connectivity density by ~64% (Fig. 7i–l), as well as decreased trabecular spacing by ~55% and structure model index by ~40% (Fig. 7k–m). Similar to NMN-treated mice, *Prx1 cre; ColA1*^*flox-stop-flox-SIRT1*^ mice presented declined adipogenesis by ~33% (Fig. 7n, o). Altogether, these results confirm that the SIRT1 is required for NMN to control osteogenesis and adipogenesis in BM MSCs.

## Discussion

Our results show that anti-aging agent NMN, can efficiently promote MSCs expansion in vivo and in vitro, not only in adult mice, but also in aged or irradiated mice. Mechanistically, SIRT1 protein upregulation plays an essential role in NMN’s regulation of bone-fat balance. While skeletal aging and osteoporosis are characterized by dysregulation of MSC fate determination^34,35^, our findings reveal a potential connection between NMN treatment and remedy in osteoporotic and aging mice.

Shortage of BM donation and extreme low ratio of MSCs in tissues necessitate in vitro expansion for clinical application of MSCs. However, most MSC expansion methods require frequent and prolonged subculturing, leading to proliferative exhaustion, senescence, compromised differentiation capacity of MSCs as well as heterogeneity resulted from spontaneous transformation^36^. Our study shows that NMN has the ability to promote MSCs expansion in vivo, which circumvented the issue of heterogeneity of in vitro culture and avoided prolonged subculturing.

Skeletal aging is featured by increased adipogenesis and reduced osteogenesis. Irradiated bone shares the similar characteristics with aged bones^31,37^. Our study indicates that NMN enhance the bone-fat balance towards the bone lineage in aged and irradiated mice, suggesting that NMN is a valuable therapy for rescuing bone loss during aging.

Previous studies have shown that SIRT1 plays an important role in regulating osteogenesis and adipogenesis in human embryonic stem cells^33^. NMN rescues age-associated susceptibility to acute kidney injury via a SIRT1-dependent pathway^38^. By using a SIRT1 inhibitor, we demonstrate that SIRT1 protein is essential for NMN to control the osteoblast and adipogenic lineage differentiation. Because of the high perinatal mortality of SIRT1 knockout mice and lack of *SIRT1*^*loxp*^ mice^39^, we were not able to study SIRT1 depletion in mice. We also noticed that *Prx1 cre; ColA1*^*flox-stop-flox-SIRT1*^ mice with SIRT1 overexpression have more robust effect on bone formation than NMN treatment. This could be due to SIRT overexpression starting at embryonic stage in *Prx1 cre; ColA1*^*flox-stop-flox-SIRT1*^ mice, whereas NMN treatment was not initiated until the adult stage.

In summary, we provide evidence for the novel role of NMN in regulating bone-fat imbalance through SIRT1 during skeletal aging. Our study establishes NMN as a promising potential therapy for MSCs expansion and rejuvenation of aged MSCs.

## Supplementary information

Figure S1

Figure S2

Figure S3

Figure S4

supplementary figure legends
