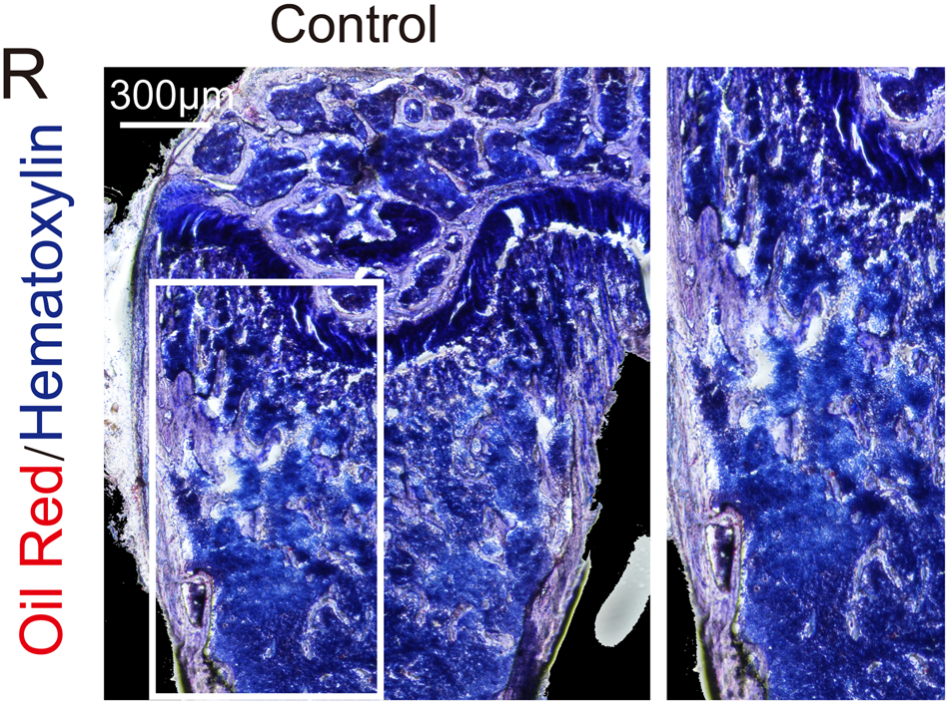# Correction: Nicotinamide mononucleotide promotes osteogenesis and reduces adipogenesis by regulating mesenchymal stromal cells via the SIRT1 pathway in aged bone marrow

**DOI:** 10.1038/s41419-025-07398-2

**Published:** 2025-03-11

**Authors:** Jie Song, Jing Li, Fangji Yang, Gang Ning, Limin Zhen, Lina Wu, Yongyuan Zheng, Qi Zhang, Dongjun Lin, Chan Xie, Liang Peng

**Affiliations:** 1 Department of Infectious Diseases, The Third Affiliated Hospital of Sun Yat-Sen University, Guangzhou, China; 2 Guangdong-Hongkong-Macau Institute of CNS Regeneration, Ministry of Education CNS Regeneration Collaborative Joint Laboratory, Jinan University, Guangzhou, China; 3 Cell-Gene Therapy Translational Medicine Research Center, The Third Affiliated Hospital of Sun Yat-Sen University, Guangzhou, China; 4 Department of Haematology, The Seventh Affiliated Hospital of Sun Yat-Sen University, Shenzhen, China

Correction to: *Cell Death and Disease* 10.1038/s41419-019-1569-2, published online 18 April 2019

Due to an oversight in figure selection, the original representative image of Figure 6R is incorrect. However, this does not affect the experimental conclusions. Therefore, it is necessary to correct Figure 6R.

Originally published figure 6R:
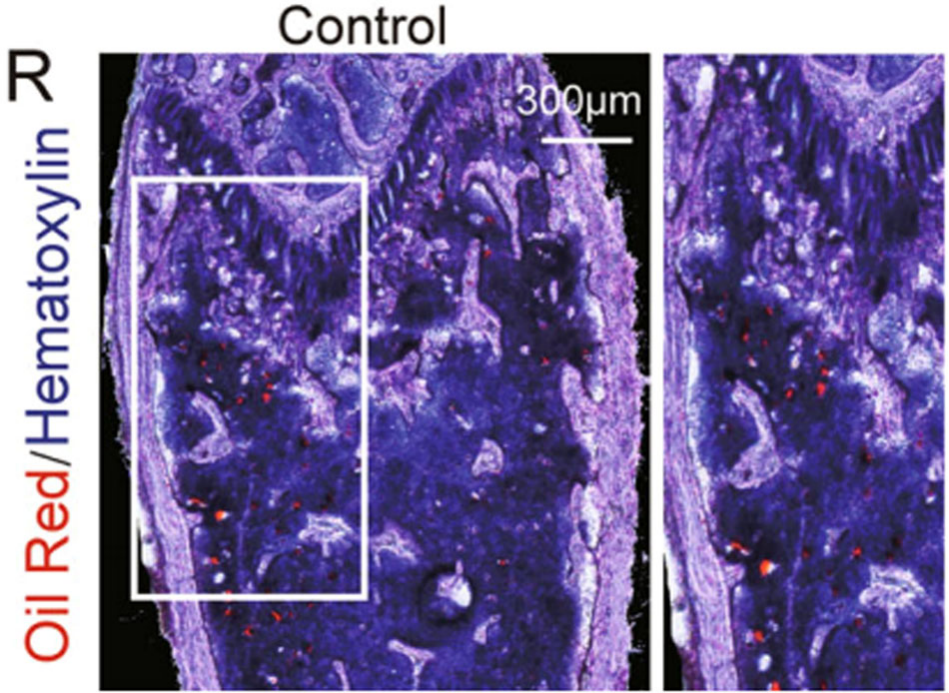


Updated figure 6R: