# Supplementary figures and images for: Nicotinamide mononucleotide promotes osteogenesis and reduces adipogenesis by regulating mesenchymal stromal cells via the SIRT1 pathway in aged bone marrow
Source: Cell Death Dis. 2019 Apr 18;10(5):336. doi: 10.1038/s41419-019-1569-2 (PMC6472410; doi:10.1038/s41419-019-1569-2)

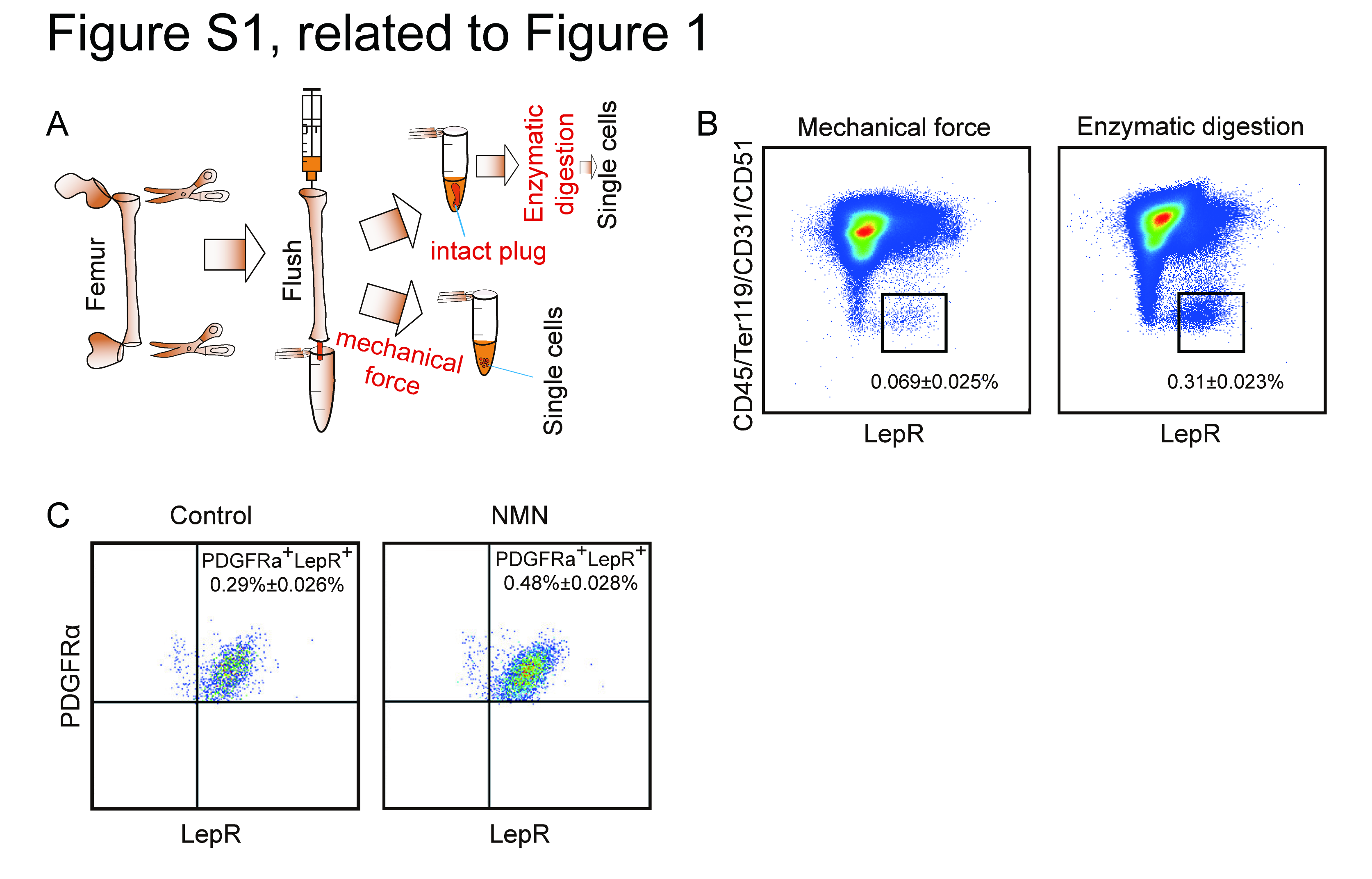

Supplement: Supplementary file 1 — Figure S1 [file 41419_2019_1569_MOESM1_ESM.tif]

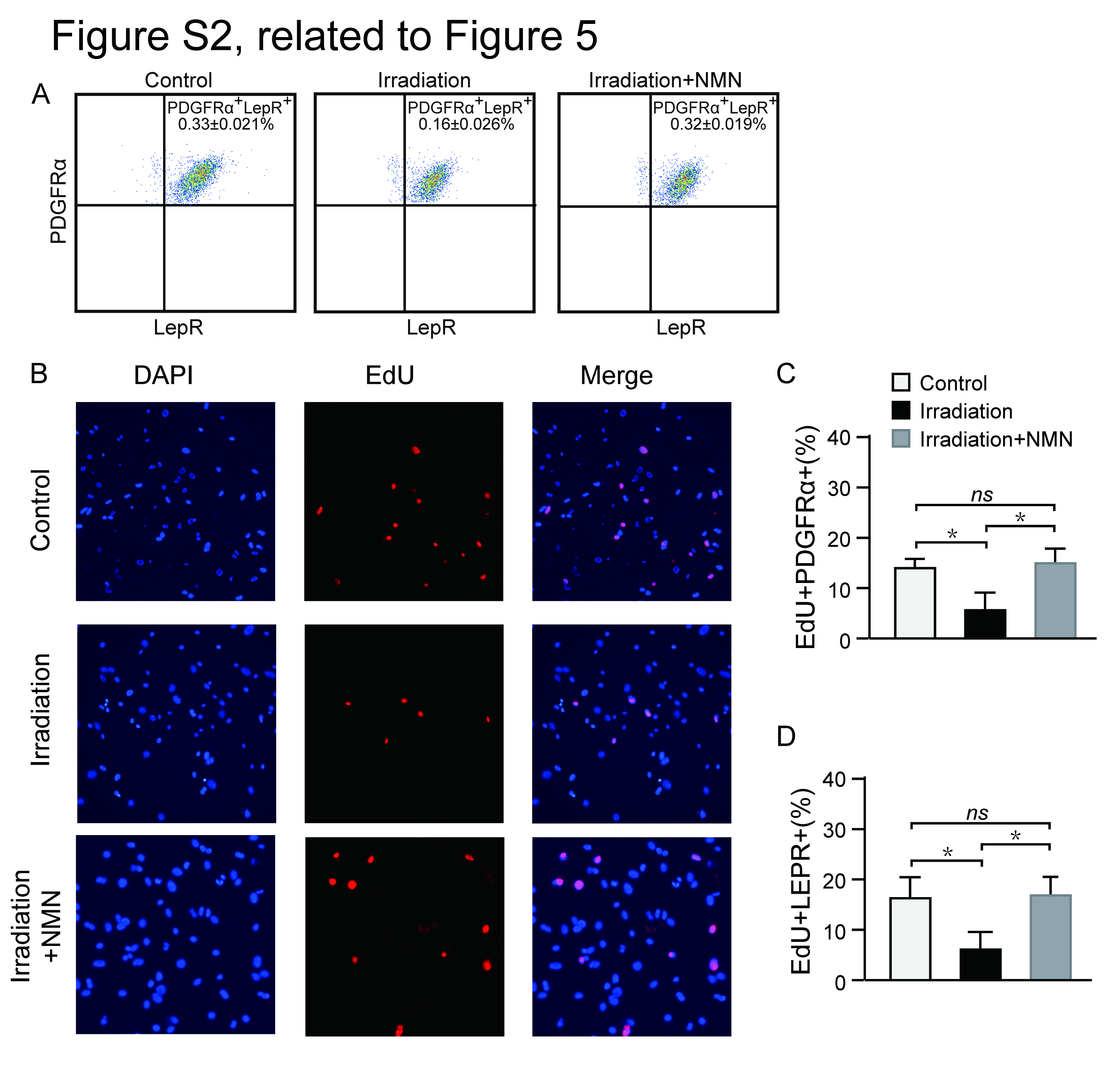

Supplement: Supplementary file 2 — Figure S2 [file 41419_2019_1569_MOESM2_ESM.tif]

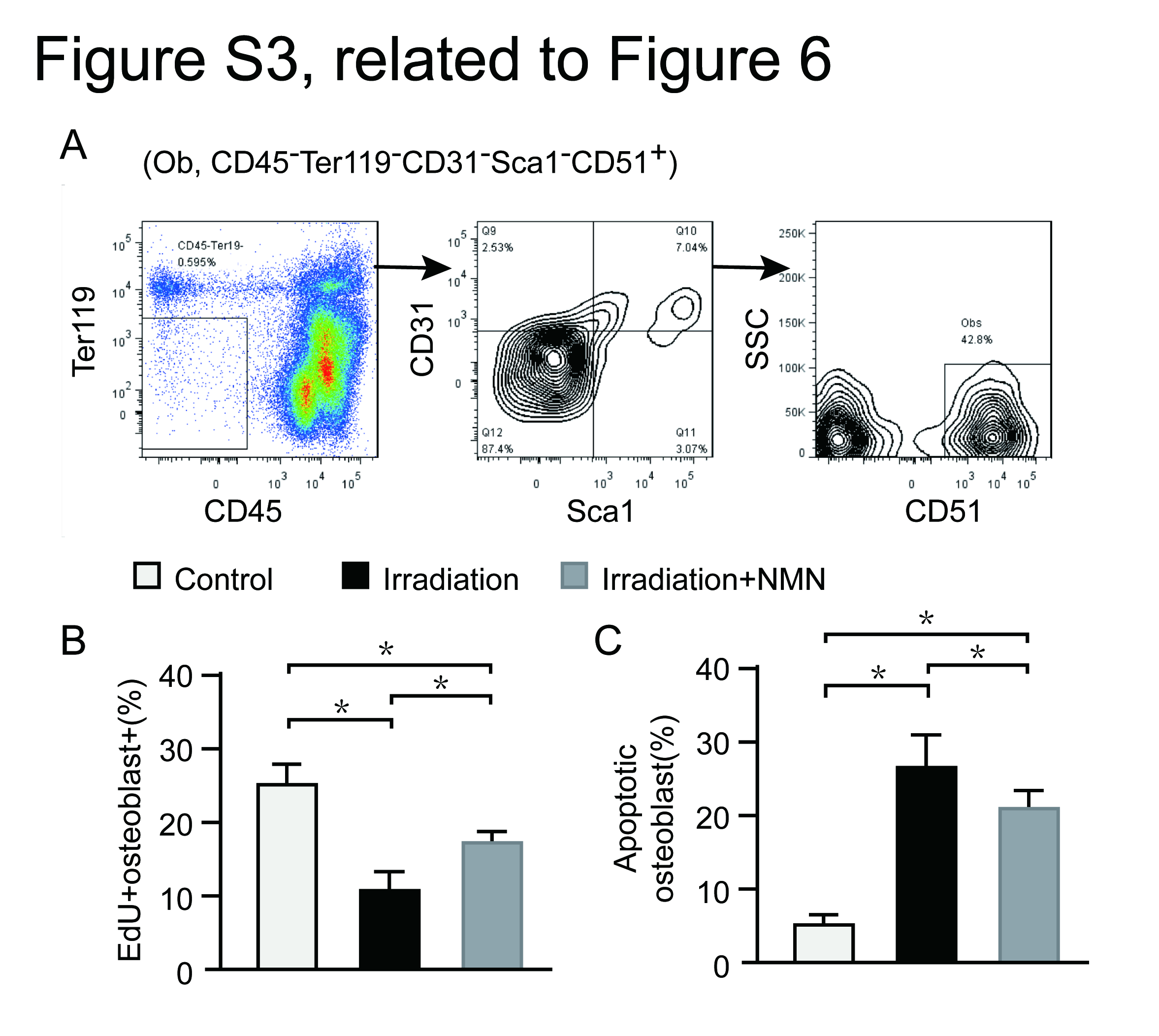

Supplement: Supplementary file 3 — Figure S3 [file 41419_2019_1569_MOESM3_ESM.tif]

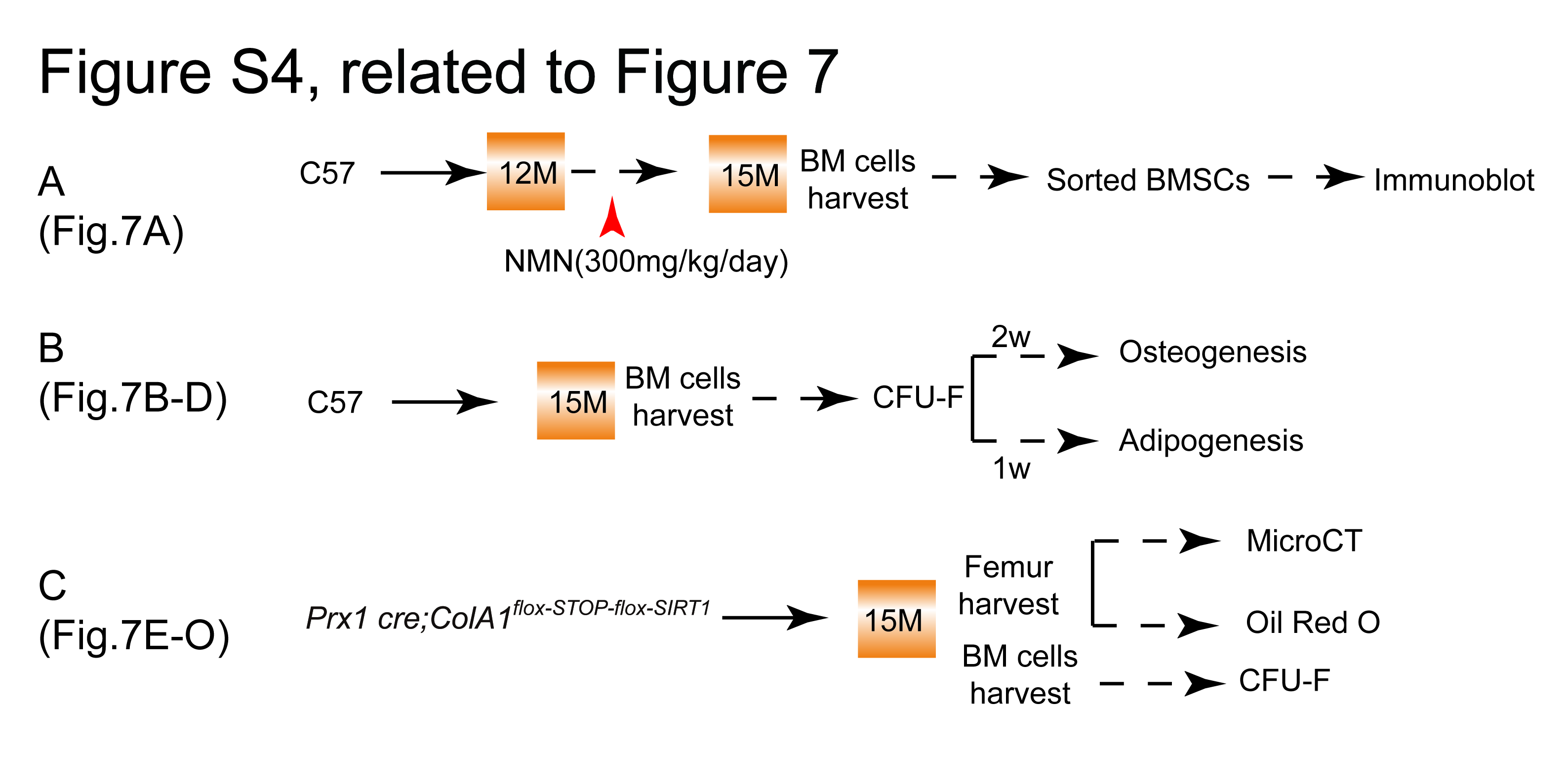

Supplement: Supplementary file 4 — Figure S4 [file 41419_2019_1569_MOESM4_ESM.tif]
