# Supplementary material for: Nicotinamide mononucleotide promotes osteogenesis and reduces adipogenesis by regulating mesenchymal stromal cells via the SIRT1 pathway in aged bone marrow
Source: Cell Death Dis. 2019 Apr 18;10(5):336. doi: 10.1038/s41419-019-1569-2 (PMC6472410; doi:10.1038/s41419-019-1569-2)
Supplement: Supplementary file 5 — supplementary figure legends [file 41419_2019_1569_MOESM5_ESM.docx]

## Fig. S1. The enzymatic digestion method yields many more MSCs than the mechanical isolation method.

(A) Schematic diagram showing the process of flushing intact marrow plugs from the long bones, followed by enzymatic digestion.

(B) Few LepR^+^ stromal cells from mechanically isolated femur bone marrow cells were detected (B, left), while LepR^+^ stromal cells from enzymatically dissociated femur bone marrow cells stained positively for LepR (B, right).

(C) Flow cytometric analysis of PDGFRα^+^LepR^+^ MSCs from enzymatically dissociated femur bone marrow cells.

## Fig. S2. NMN promotes MSCs proliferation after irradiation.

1. Flow cytometric analysis of PDGFRα^+^LepR^+^ MSCs from enzymatically dissociated femur bone marrow cells.
2. Representative images showing EdU-labelled MSCs sorted from the bone marrow. All cell nuclei show blue fluorescence indicative of DAPI staining (original magnification, 20×).

(C-D) ﻿The relative ratio of EdU-positive cells in PDGFRα^+^ MSCs (C) or LepR^+^ MSCs (D).

## Fig. S3. NMN might have direct effect on osteoblast cells in irradiated mice.

(A) Flow cytometric gating of osteoblasts (CD45^–^Ter119^–^CD31^–^Sca1^–^CD51^+^).

(B) The relative ratio of EdU-positive cells in osteoblasts sorted from the bone marrow.

(C) The relative ratio of caspase-3/7-positive cells to total osteoblasts sorted from the bone marrow.

## Fig. S4. Schematic diagram showing NMN administration and downstream analyses.
